# Navigating Perinatal Challenges: A Comprehensive Review of Hepatitis B Viral Infection and Pregnancy Outcomes

**DOI:** 10.7759/cureus.59028

**Published:** 2024-04-25

**Authors:** Aditi Singh Thakur, Surekha Tayade, Nitish Batra, Neha Sethi, Arpita Jaiswal

**Affiliations:** 1 Obstetrics and Gynecology, Jawaharlal Nehru Medical College, Datta Meghe Institute of Higher Education and Research, Wardha, IND

**Keywords:** public health interventions, antiviral therapy, perinatal outcomes, vertical transmission, pregnancy, hepatitis b

## Abstract

Hepatitis B viral infection poses a significant challenge during pregnancy, as the risk of vertical transmission carries serious consequences for both maternal and neonatal well-being. This comprehensive review delves into the intricacies surrounding hepatitis B infection during the perinatal period, shedding light on its impact on pregnancy outcomes and stressing the necessity of addressing it within the broader framework of perinatal care. By scrutinizing current evidence, diagnostic methodologies, management techniques, and preventive measures, this review emphasizes the urgent need for enhanced screening protocols, timely interventions, and augmented public health initiatives. Notably, key findings underscore the elevated likelihood of chronic hepatitis B virus (HBV) infection in infants and its enduring implications for the health of both mothers and newborns. The imperative call to action advocates for a multifaceted approach, engaging healthcare professionals, policymakers, and public health agencies to optimize strategies for management and prevention, thereby striving for improved outcomes for pregnant women and their infants affected by hepatitis B viral infection.

## Introduction and background

Perinatal challenges encompass a spectrum of health issues and complications that emerge during the critical phases of pregnancy, childbirth, and the postnatal period [1]. These challenges affect the mother and the newborn, and their impact can extend beyond the immediate perinatal period, shaping the long-term health trajectories of both individuals. From maternal conditions such as gestational diabetes and hypertensive disorders to neonatal complications like prematurity and congenital disabilities, perinatal challenges present a complex array of medical concerns that require careful management and intervention [2].

Among the multitude of perinatal challenges, hepatitis B viral infection stands out as a particularly significant threat to maternal and fetal health. Hepatitis B is a highly infectious liver disease caused by the hepatitis B virus (HBV), and it can be transmitted vertically from an infected mother to her child during pregnancy or childbirth [3]. This mode of transmission poses a substantial risk to the newborn, as it can result in chronic HBV infection, which may lead to severe liver-related complications later in life, including liver cirrhosis and hepatocellular carcinoma (HCC) [4]. Addressing HBV infection during pregnancy is paramount for several reasons. Firstly, preventing vertical transmission of HBV is essential for safeguarding the health and well-being of the newborn, as chronic HBV infection acquired early in life carries a higher risk of long-term complications [5]. Secondly, maternal health can also be adversely affected by HBV infection during pregnancy, with increased risks of liver dysfunction and exacerbation of existing liver disease. By implementing effective strategies for screening, diagnosis, and management of HBV infection in pregnant women, healthcare providers can mitigate these risks and optimize outcomes for both mothers and infants [6].

The purpose of this comprehensive review is to provide an in-depth analysis of the challenges posed by hepatitis B viral infection during pregnancy and its impact on pregnancy outcomes. By synthesizing current evidence and exploring diagnostic, management, and prevention strategies, this review aims to enhance understanding of the complexities surrounding HBV infection in the perinatal period and inform clinical practice and public health interventions to improve maternal and neonatal health.

## Review

Epidemiology of hepatitis B

Global Prevalence

The global prevalence of chronic HBV infection has been a significant concern for public health. As of 2019, it was estimated that approximately 4.1% of the global population of all ages, totaling around 316 million individuals worldwide, were affected by chronic HBV infection [7]. Over time, there has been a decreasing trend in the prevalence of HBV infection, with a notable decline of 31.3% observed between 1990 and 2019. This decline is particularly pronounced in children under five, with a remarkable 76.8% decrease in prevalence [7]. However, despite these improvements, HBV-related diseases still led to a substantial number of global deaths, with an estimated 555,000 deaths attributed to HBV in 2019 [7]. In the United States, the prevalence of any past or present HBV infection was reported to be 4.3% from 2015 to 2018. This represents a decreasing trend from 5.7% in 1999-2002 to 4.3% in 2015-2018 [8]. Furthermore, there has been an increase in the prevalence of hepatitis B vaccination in the United States, rising from 12.3% in 1999 to 25.2% in 2018. This indicates progress in vaccination efforts to combat HBV infection [8]. These findings underscore the ongoing endeavors to alleviate the global burden of HBV infection through vaccination programs and public health interventions. They emphasize the importance of continued surveillance and strategic initiatives to achieve the ambitious goal of eliminating viral hepatitis by 2030.

Modes of Transmission

HBV transmission from an infected mother to her newborn, known as perinatal transmission, is a significant risk, mainly when the mother tests positive for hepatitis B surface antigen (HBsAg) and has a high serum HBV-DNA level. Without the proper administration of prophylaxis, newborns of HBsAg-positive mothers face a substantial risk of becoming infected. This condition often progresses to chronic HBV infection, underscoring the importance of preventive measures in the perinatal period to safeguard the health of the infant [9]. Another prevalent mode of HBV transmission is through direct blood exposure. This can occur via the use of contaminated needles, engagement in medical procedures that utilize unsterile equipment, and any activity involving direct contact with infected blood. Practices such as needle sharing among individuals who use injection drugs, as well as tattooing, piercing, and similar procedures that involve blood contact, are notable contributors to the spread of HBV. The risks associated with these activities highlight the critical need for strict adherence to safe medical practices and implementing harm-reduction strategies to prevent HBV transmission [9].

**Figure 1:**
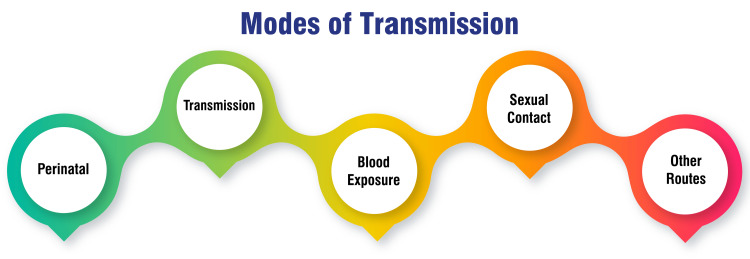
Modes of transmission The image is created by the corresponding author.

Sexual contact also serves as a significant route for the spread of HBV, primarily among unvaccinated individuals who have multiple sexual partners. In regions where HBV prevalence is low, sexual transmission plays a significant role in the incidence of HBV infection among adolescents and adults. This points to the necessity of promoting vaccination and sexual health education as part of comprehensive strategies to reduce the transmission of HBV through sexual contact [9]. Additionally, HBV can be transmitted through the use of contaminated personal items, such as toothbrushes and razors, and the sharing of sharp instruments. However, it is crucial to recognize that HBV is not spread through casual contact, including sneezing, coughing, or sharing meals. Understanding these various modes of transmission is vital for public health efforts focused on reducing the spread of HBV. Emphasizing the importance of vaccination, ensuring the use of sterile medical equipment, and fostering awareness about safe practices can significantly decrease the health risks associated with HBV transmission [9]. Figure 1 shows the modes of transmission.

Impact on Pregnancy Outcomes

The influence of HBV infection on pregnancy outcomes constitutes a significant concern, with research illuminating the potential connections between a maternal HBV carrier state and various adverse outcomes. Studies have demonstrated that pregnant women who are HBV carriers might be at an elevated risk for several adverse pregnancy outcomes, including miscarriage, preterm birth, and gestational diabetes, among others [10-12]. A particular prospective cohort study highlighted a notable correlation between maternal HBV carrier status and an increased frequency of miscarriage, even when adjustments were made for sociodemographic factors and obstetric complications [12]. Moreover, a comprehensive meta-analysis has pointed out that HBV infection during pregnancy could heighten the risk for gestational diabetes, preterm delivery, and preeclampsia, underscoring the critical importance of meticulous monitoring and management of HBV-infected pregnant women to enhance pregnancy outcomes [11].

In addition, a population-based cohort study carried out in the United States found that while HBV-infected pregnant women were predominantly Asian and born outside of the United States, their risk for adverse pregnancy outcomes was not significantly higher in comparison to their HBV-negative counterparts, with the exception being a diminished risk of having infants small for gestational age [10]. The research surrounding HBV infection and pregnancy outcomes presents a complex picture, indicating an association between HBV infection in pregnant women and an increased risk of adverse events such as miscarriage, preterm birth, and gestational diabetes. These women must receive thorough monitoring, early detection, and appropriate management to alleviate these risks and ensure the best possible pregnancy outcomes.

HBV in pregnancy

Vertical Transmission Risk

Vertical transmission of HBV represents a notable risk, particularly in the absence of preventive measures. The likelihood of vertical transmission is most pronounced in mothers who test positive for both HBsAg and hepatitis B e-Antigen (HBeAg), with transmission rates estimated to be between 70% and 90%. Conversely, for mothers who are only HBsAg-positive but HBeAg-negative, the risk diminishes from 10% to 40% [13]. Nonetheless, the application of appropriate prophylaxis, such as administering hepatitis B immune globulin (HBIG) alongside the hepatitis B vaccine to newborns of HBsAg-positive mothers within the first 24 hours after birth, can significantly lower the risk of vertical transmission [13,14]. Despite these interventions, a residual risk of approximately 3% remains, especially in elevated maternal viral load [13].

The presence of maternal viremia, denoted by high levels of circulating HBV DNA or the presence of HBsAg and HBeAg, is a pivotal factor that increases the risk of HBV's vertical transmission. Situations that facilitate maternal-fetal microtransfusions of HBV-infected maternal blood can heighten the risk of transmission. This risk can manifest intrauterine, during labor, or at the time of delivery [13]. Therefore, implementing proper management and prevention strategies, including antiviral therapy during the third trimester of pregnancy and immunoprophylaxis for newborns, is critical in mitigating the risk of vertical transmission. These measures play a crucial role in enhancing outcomes for infants born to HBV-infected mothers [15].

Maternal Factors Influencing Transmission

The transmission of the HBV during pregnancy is significantly influenced by maternal factors, particularly the mother's HBsAg and HBeAg status. Research indicates that the likelihood of perinatal HBV infection in newborns is directly impacted by whether the mother tests positive for HBsAg and her HBeAg status. Specifically, infants born to HBsAg-positive mothers who also have a positive HBeAg status face a considerably higher risk of contracting perinatal HBV infection compared to infants born to mothers with a negative HBeAg status [16]. In the absence of prophylactic measures, the risk of perinatal HBV infection for infants of HBsAg-positive mothers varies dramatically, ranging from less than 10% to as much as 70%-90%, dependent on the HBeAg status of the mother [16].

To prevent the perinatal transmission of HBV, several interventions are recommended, including the universal screening of pregnant women for HBV, the vaccination of infants born to HBV-positive mothers within the first 24 hours after birth, and ensuring the completion of the HBV vaccination series for these infants by the age of 18 months [17]. For mothers who test positive for HBV, case management throughout pregnancy is critical. This involves testing for HBV DNA viral load and referring these mothers to specialized care for counseling and the provision of appropriate medical interventions to minimize the risk of perinatal transmission [17]. Maternal factors, such as HBsAg and HBeAg status, are paramount in assessing the risk of perinatal HBV transmission. The implementation of thorough screening, effective vaccination, and comprehensive case management strategies is vital for the prevention of HBV transmission from mother to child during pregnancy and at the time of childbirth.

Impact on Maternal Health

The repercussions of HBV infection on maternal health, especially during pregnancy, are profound. HBV infection among pregnant women carries significant risks for both the mother and the infant. In the absence of postexposure prophylaxis, the likelihood of infants born to HBV-infected mothers contracting HBV escalates to as high as 90%, with these infections often progressing to chronic states. This scenario poses a severe risk of liver-related complications for the child, with approximately a quarter of these infants facing the possibility of liver-related death later in life [18]. Moreover, being an HBV carrier is linked with adverse pregnancy outcomes, including an elevated risk of miscarriage and preterm birth in natural pregnancies [18,19].

Studies have documented that maternal HBV infection may amplify the risk of adverse outcomes such as miscarriage, preterm birth, and gestational diabetes. This underscores the critical need for diligent monitoring and specialized care for pregnant women diagnosed with HBV [12,18]. Interestingly, while HBV infection does not show significant associations with specific pregnancy outcomes in assisted reproductive technologies, such as freeze-thaw embryo transfer (FET), the risk of miscarriage remains higher in HBV carriers. This observation underscores the importance of careful surveillance and management throughout pregnancy [20]. The data collectively emphasize the necessity for rigorous health protocols to safeguard pregnant women with HBV infection and mitigate risks to maternal and infant health.

Diagnostic and screening strategies

Antenatal Screening Protocols

Sickle cell and thalassemia screening: This initiative underscores the commitment to early detection of sickle cell disease and thalassemia, offering screenings typically by the 10th week of pregnancy. The prompt timeline aims to furnish at-risk women with prenatal diagnoses by the 12th week, thereby facilitating informed decision-making. This program is pivotal in educating parents about screening, interpreting results, and setting realistic expectations for their baby's health outcomes [21].

Group B streptococcus (GBS) screening: Recognizing the potential severity of GBS transmission during childbirth, this screening is conducted between the 36th and 38th weeks of pregnancy. A positive test result triggers the administration of antibiotics during labor, a preventive measure designed to shield the fetus from infection. This screening is crucial for mitigating the risk of GBS-related complications in newborns [22].

Genetic testing for birth defects: Early pregnancy is critical for assessing congenital disabilities' risk. While screening tests provide risk estimations, confirmatory diagnostics are necessary for a definitive assessment. The choice to undergo these tests reflects a personal decision, often driven by the desire for advanced knowledge of potential health issues, allowing families to prepare for various outcomes [22].

Infectious disease screening: Routine screening for human immunodeficiency virus (HIV), hepatitis B, hepatitis C, and syphilis is advocated for every pregnancy. This crucial step enables access to treatments and medical interventions, significantly reducing perinatal transmission risk. Early detection through screening is fundamental in safeguarding the health of both the mother and the infant [23].

**Figure 2:**
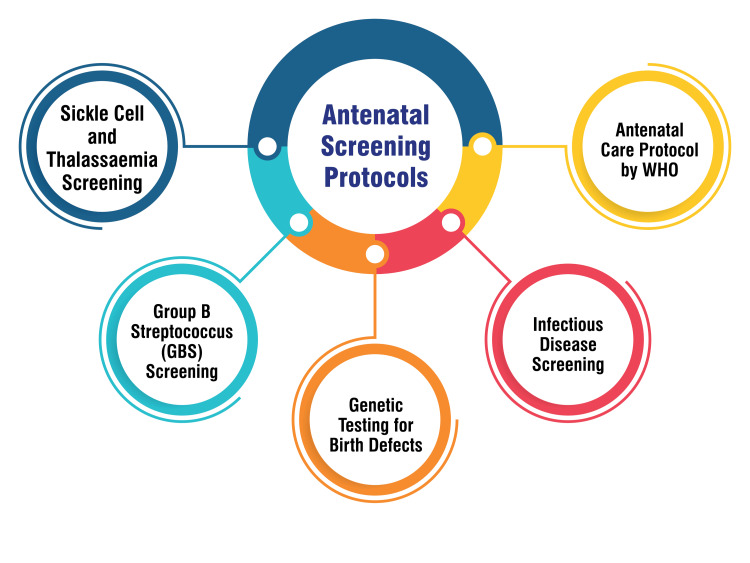
Antenatal screening protocols The image is created by the corresponding author.

Antenatal care (ANC) protocol by the World Health Organization (WHO): The WHO’s emphasis on ANC highlights the global recognition of its importance in maternal and child health. The WHO outlines specific visit schedules throughout pregnancy to detect complications early and administer appropriate care. This guideline is instrumental in promoting health and well-being during pregnancy, culminating in positive outcomes for both mother and baby [24]. Figure 2 shows antenatal screening protocols.

Diagnostic Tests for HBV Infection During Pregnancy

The evaluation for HBV infection in pregnant women predominantly relies on blood tests to identify specific indicators of the virus. The primary method for detecting maternal HBV infection is the HBsAg test [25-27]. This test is recommended to be conducted during the initial prenatal visit for every pregnant woman, irrespective of her vaccination status against HBV or previous negative test outcomes [27]. Furthermore, the US Preventive Services Task Force (USPSTF) advocates for the screening of all pregnant women for HBsAg as a means to pinpoint those who are at an elevated risk for perinatal transmission of the virus [27]. Additionally, it is advised that laboratories conducting HBsAg testing should note the pregnancy status of women undergoing this test, as this information is vital for reporting and surveillance [28]. Healthcare providers and public health departments are instrumental in ensuring accurate laboratory reporting of the pregnancy status of women diagnosed with HBV. This includes requesting HBsAg tests be marked as "prenatal" and communicating a patient's pregnancy status to laboratories to guarantee that confirmatory testing is conducted on all positive findings [28].

Challenges and Limitations

Diagnosing HBV infection presents formidable challenges, impacting the accurate identification and management of affected individuals. Despite the global prevalence of HBV, a significant portion of the population remains undiagnosed, with an estimated 90% of infected individuals going unrecognized [29]. This widespread lack of diagnosis erects barriers to effective treatment and prevention efforts, hampering endeavors to curb the spread of HBV and its associated complications. A primary obstacle in HBV diagnosis stems from the reliance on conventional methods, such as tests based on HBsAg, which may not capture all phases of HBV infection, including occult HBV infection (OBI) [2]. Factors like the window period, immune control, and OBI pose diagnostic challenges as they may evade detection by standard tests, leading to underdiagnosis and potential transmission risks. Additionally, identifying HBV S variants and immune complex-bound HBsAg remains elusive, necessitating the development of a more sensitive and standardized testing protocols to enhance diagnostic precision [29].

Moreover, the multifaceted nature of HBV infection phases, encompassing immune-tolerant, immune activation, reactivation, and remission, further complicates diagnosis. Accurately distinguishing between these phases and monitoring disease progression is vital for guiding treatment decisions and optimizing patient outcomes. The current limitations in diagnostic tools underscore the imperative for a comprehensive approach to augment HBV diagnosis and achieve the WHO's objective of eliminating hepatitis B by 2030 [30]. Addressing the challenges and constraints in HBV diagnosis necessitates advancing and deploying innovative diagnostic platforms, novel marker assays, and sophisticated testing methodologies to effectively detect all HBV infection phases. By enhancing awareness, bolstering case identification, refining surveillance strategies, and optimizing treatment protocols, healthcare systems can surmount these obstacles and make strides toward eliminating HBV as a public health threat.

Management approaches

Antiviral Therapy During Pregnancy

Administering antiviral therapy during pregnancy is pivotal in managing chronic HBV infection to mitigate the risk of perinatal transmission and promote favorable outcomes. Tenofovir (TDF) and entecavir (ETV) are the recommended first-line oral antiviral therapies for pregnant women with HBV due to their efficacy and safety profiles compared to alternatives like lamivudine (LAM) and telbivudine (LdT), which carry higher rates of resistance and potential cross-resistance with other treatments [31]. Initiating antiviral therapy during the third trimester, specifically starting from week 28 of pregnancy until delivery, is critical for reducing the risk of perinatal HBV transmission [32]. Pregnant women with elevated viral loads, particularly those positive for HBeAg, may derive particular benefit from TDF treatment during the final trimester to prevent transmission to the newborn [32]. It is imperative to carefully weigh the risks and benefits of commencing antiviral therapy during pregnancy, considering its potential impact on both maternal and fetal health [31].

Continuous antiviral therapy throughout pregnancy has demonstrated efficacy in preventing mother-to-child transmission (MTCT) of HBV, especially in scenarios where immunoprophylaxis may prove insufficient, such as in infants born to highly viremic mothers [33]. This ongoing therapy significantly diminishes the risk of HBV transmission to the offspring and constitutes a crucial measure in managing chronic HBV infection during pregnancy [33]. Antiviral therapy during pregnancy plays a vital role in managing chronic HBV infection to shield both the mother and the newborn from the associated transmission risks. Initiating appropriate antiviral treatment, monitoring viral loads, and adhering to recommended guidelines are essential to ensure the safety and well-being of both the pregnant woman and the child.

Immunoprophylaxis for Neonates

HBV prophylaxis: Postexposure prophylaxis for infants born to HBsAg-positive mothers involves a regimen comprising 0.5 mL of HBIG and a hepatitis B vaccine (HepB). Following this initial administration, two additional doses of HepB are administered at one to two months and six months of age. This comprehensive approach has been demonstrated to prevent HBV infection in 85% to 95% of infants [34]. Notably, studies have revealed that the protective efficacy rate of HBV vaccination in infants born to carrier mothers falls within the range of 75.3% to 89.7% [35]. This underscores the paramount importance of vaccination in mitigating chronic HBV carrier rates among newborns, highlighting the pivotal role of proactive preventive measures in safeguarding infant health and preventing the transmission of HBV.

Respiratory syncytial virus (RSV) prophylaxis: Palivizumab immunoprophylaxis is a critical intervention to prevent severe RSV infections, particularly in high-risk infants. Such high-risk groups include infants with congenital heart disease, Down syndrome, or cystic fibrosis [36]. However, using palivizumab prompts cost-effectiveness evaluations to ascertain its economic viability in healthcare settings. Various cost-effectiveness analyses have been conducted, yielding diverse conclusions regarding the cost-effectiveness of palivizumab across different risk groups and hospitalization scenarios [37]. These evaluations inform healthcare decision-makers and practitioners about the potential economic implications of implementing palivizumab immunoprophylaxis, facilitating informed decision-making and resource allocation strategies to optimize infant health outcomes while considering healthcare budgetary constraints and priorities.

Monitoring and Follow-Up Protocols

Testing protocols: The testing protocols for managing HBV infection during pregnancy are crucial for ensuring early detection and appropriate management. It is recommended that all pregnant women undergo screening for HBsAg to identify any potential HBV infection promptly [38]. Additionally, HBsAg-positive pregnant women should undergo further testing for HBeAg and HBV DNA. These additional tests help assess the risk of transmission to the infant and determine the infectivity level, enabling healthcare providers to tailor interventions accordingly [27].

Education and counseling: Effective education and counseling are essential components of comprehensive care for pregnant women diagnosed with HBV infection. Healthcare professionals should provide detailed information about disease management strategies and plan ongoing care for these women [38]. Furthermore, education should be extended to family members and close contacts, emphasizing the importance of testing and appropriate preventive measures to prevent transmission [38].

Prevention of transmission: Preventive measures are pivotal in mitigating the risk of vertical transmission of HBV from mother to child. Infants born to HBsAg-positive mothers should receive immediate postnatal intervention, including a combination of HBIG and the first dose of the HepB, followed by completion of the vaccine series [38]. In cases of high viral loads, referral to a specialist is recommended to discuss the potential use of TDF between 28 and 32 weeks gestation to reduce the risk of perinatal transmission further [38].

Postpartum monitoring: Close monitoring during the postpartum period is essential to detect any potential flares of hepatitis B activity, which may occur due to immunological changes. Women with high viral loads and HBeAg positivity are particularly at risk of postpartum flares, necessitating vigilant monitoring to ensure timely intervention and management [39].

**Figure 3:**
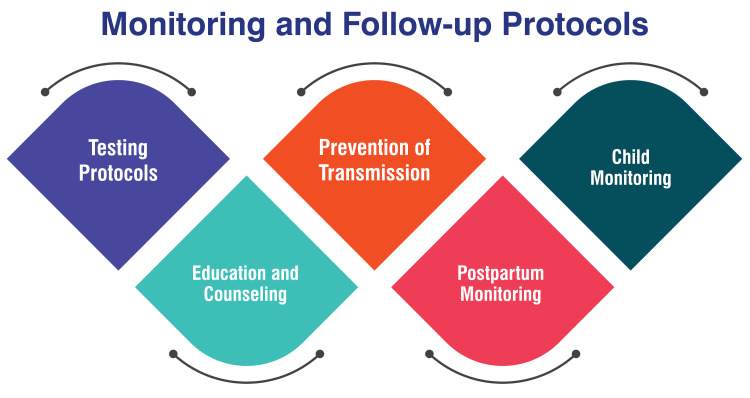
Monitoring and follow-up protocols The image is created by the corresponding author.

Child monitoring: Regularly monitoring children born to HBsAg-positive mothers is critical for the early detection and management of HBV infection. Children should undergo testing for HBsAg and anti-HBs at 9-12 months of age to assess their infection status [38]. For those diagnosed with chronic hepatitis B (CHB), annual monitoring with liver function tests, HBV serology, and viral load assessments is recommended [38]. By adhering to these monitoring and follow-up protocols, healthcare providers can effectively manage HBV infection during pregnancy, reduce the risk of transmission, and ensure the well-being of both the mother and the child. Figure 3 shows the monitoring and follow-up protocols.

Pregnancy outcomes in HBV-infected women

Maternal Complications

Miscarriage: The association between HBV carrier status and an increased risk of miscarriage among pregnant women has been substantiated by research, emphasizing the necessity for vigilant surveillance and monitoring [12]. Studies have revealed that pregnant women with HBV carrier status are more prone to experiencing miscarriages compared to those without HBV infection. This underscores the importance of early detection and continuous monitoring throughout pregnancy to address potential risks and ensure optimal maternal and fetal health outcomes.

Preterm birth: Chronic HBV infection during pregnancy has been correlated with a heightened risk of preterm birth, as well as gestational diabetes, preeclampsia, and other adverse outcomes [12,40,41]. Research indicates a higher prevalence of preterm delivery among women with chronic HBV infection, underscoring the critical significance of managing HBV infection during pregnancy to mitigate these risks [41]. Given the potential complications associated with preterm birth, including neonatal morbidity and mortality, it is essential to implement measures to manage HBV infection and optimize pregnancy outcomes effectively.

Gestational diabetes: Maternal HBV carrier status has been identified as a risk factor for gestational diabetes, necessitating close monitoring and management of diabetes during pregnancy in HBV-infected women [12,41]. Studies have demonstrated an elevated risk of gestational diabetes among pregnant women with HBV infection compared to those without, highlighting the importance of comprehensive care and monitoring to mitigate the adverse effects of coexisting conditions on maternal and fetal health.

**Figure 4:**
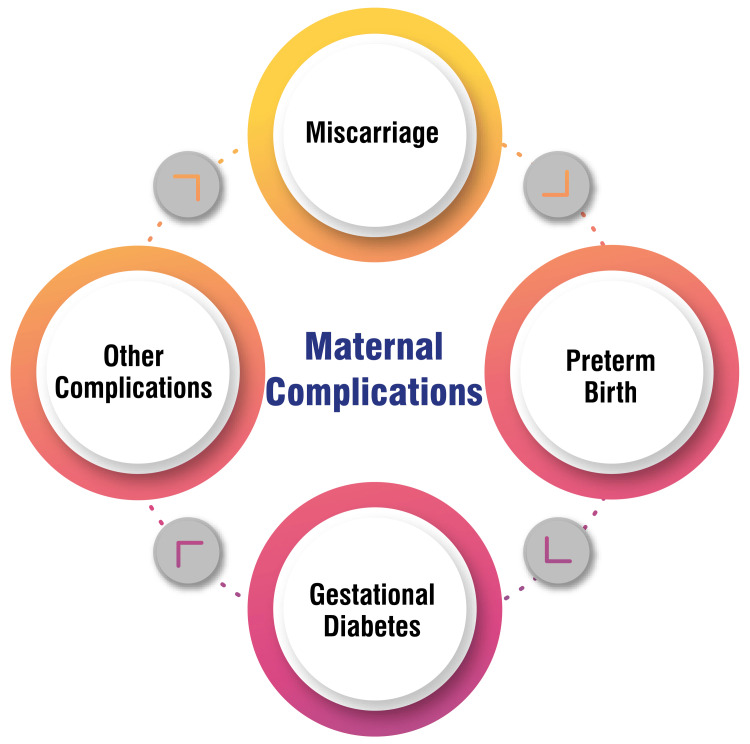
Maternal complications The image is created by the corresponding author.

Other complications: HBV infection during pregnancy may lead to various obstetric complications, including pregnancy-induced hypertension (PIH) syndrome, postpartum hemorrhage, and intrahepatic cholestasis of pregnancy (ICP) [41]. Additionally, maternal chronic HBV infection has been implicated in affecting trophoblast cells, which play a crucial role in placental development, potentially leading to further complications [41]. These findings underscore the multifaceted impact of HBV infection on pregnancy and the importance of comprehensive management strategies to address potential complications and ensure optimal outcomes for both the mother and the child. Figure 4 shows maternal complications.

Fetal and Neonatal Outcomes

Research investigating the repercussions of maternal HBV infection on fetal and neonatal outcomes has yielded invaluable insights into the risks associated with HBV during pregnancy. Numerous studies have underscored that HBV infection during pregnancy can lead to adverse outcomes, including an elevated risk of preterm birth, gestational diabetes, miscarriage, and other adverse pregnancy outcomes [10,11,19,20,40]. A comprehensive review emphasized the significant correlation between maternal HBV serostatus and miscarriage and preterm birth during natural pregnancies, indicating the potential for adverse pregnancy outcomes in women infected with HBV [20]. Moreover, a meta-analysis elucidated that chronic HBV infection during pregnancy may heighten the risk of gestational diabetes, preterm delivery, preeclampsia, and eclampsia, underscoring the severity of HBV infection's impact on pregnancy outcomes [11,40]. Additionally, a population-based cohort study conducted in the United States revealed that HBV-infected pregnant women exhibited a lower risk of infants being small for gestational age. However, their risks for other adverse outcomes did not significantly differ from those of HBV-negative women [10]. This study illuminates the intricate interplay between HBV infection and pregnancy outcomes, emphasizing the imperative for further research to comprehensively grasp the implications of HBV on fetal and neonatal health.

Long-Term Implications for Offspring

The long-term consequences for offspring of individuals with chronic HBV infection carry significant implications, particularly among children and adolescents. Despite the relatively mild course of chronic HBV infection during childhood and adolescence, HBV carriers face a lifetime risk of developing HCC of up to 25% [42]. Understanding the natural progression of chronic HBV infection in children is paramount as it can eventually lead to severe liver complications such as liver cirrhosis or HCC [43]. In regions where HBV endemicity is high, perinatal transmission from highly infectious mothers to their neonates serves as a standard route of HBV infection, with the age at which HBV infection occurs significantly influencing the outcome. Infants born to mothers positive for both HBeAg and HBsAg exhibit a high likelihood of developing chronic HBV infection, underscoring the critical importance of early detection and management to avert long-term complications [43]. Moreover, chronic HBV infection in children may necessitate specific treatment approaches, given that conventional treatments like interferon-α and LAM have limitations concerning tolerability and the emergence of drug-resistant HBV strains [43]. Comprehending the natural trajectory of chronic HBV infection in children and implementing tailored clinical management strategies are imperative to mitigate the enduring implications for offspring and diminish the risk of severe liver-related complications in adulthood.

Strategies for prevention and intervention

Vaccination Programs

Universal immunization: A fundamental intervention in thwarting MTCT of HBV and curtailing early childhood transmission is universal immunization of infants against hepatitis B. The WHO advocates for the universal immunization of infants with a minimum of three doses of the HepB, with the initial dose ideally administered within 24 hours of birth [44].

Peripartum antiviral prophylaxis: Pregnant women identified with HBV infection are advised to undergo TDF prophylaxis from the 28th week of pregnancy until at least the time of birth. This measure aims to prevent further transmission of HBV during pregnancy and delivery. Coupled with infant HBIG prophylaxis shortly after birth, it enhances protection against MTCT of HBV [44].

Adult vaccination: The Advisory Committee on Immunization Practices (ACIP) recommends HepB vaccination for adults aged 19-59 years with risk factors for HBV infection. These factors include exposure through sexual contact, blood exposure, and other specific groups. Vaccinating adults, particularly those at heightened risk, is pivotal in curbing the spread of HBV and alleviating the disease burden in the adult populace [45].

Global elimination targets: The WHO has established ambitious targets for eradicating hepatitis B as a public health menace by 2030. These objectives entail reducing the prevalence of HBsAg to less than 0.1% in children aged five years. Achieving these targets necessitates a comprehensive approach, encompassing heightened coverage of timely birth-dose HBV vaccination, substantial enhancements in vaccine coverage, and broadening case-finding efforts [44]. Through the implementation of extensive vaccination programs targeting both infants and adults, alongside tailored interventions for pregnant women, substantial strides can be made in curtailing HBV transmission, thwarting chronic infections, and ultimately advancing toward the global eradication of hepatitis B as a significant public health threat.

Public Health Initiatives

Inclusion health agenda: As the global health community acknowledges the significant threat posed by HBV, there is a concerted effort to integrate HBV into the inclusion health agenda. This agenda prioritizes marginalized and underserved populations, including migrants, homeless individuals, and substance users, who encounter barriers to accessing healthcare. By addressing the unique needs of these populations, public health initiatives can better target HBV interventions, ultimately working to diminish health inequities [46].

Prevention of MTCT: Mitigating MTCT of HBV stands as a pivotal component of public health strategies. This encompasses several vital interventions, including universal immunization of newborns against HBV, administering peripartum antiviral prophylaxis for HBsAg-positive pregnant women, and ensuring timely birth-dose vaccination to curtail vertical transmission events and shield infants from HBV infection [44,47].

Vaccination programs: Universal immunization programs tailored for infants, children, and adults at risk of HBV infection constitute indispensable public health interventions. Maintaining high coverage rates of HepB vaccination, which encompasses timely birth-dose vaccination and completion of the vaccine series, is paramount in diminishing the prevalence of HBV and averting new infections [44].

Policy recommendations: Public health policies, such as those advocated by the ACIP, offer guidance on various aspects, including screening pregnant women for HBsAg, administering HepB, and HBIG to newborns, and vaccinating populations at heightened risk. These policies are geared toward bolstering prevention, screening, and vaccination endeavors to alleviate the burden of HBV infection [48].

Education and Awareness Campaigns

Healthcare worker (HCW)-led educational model in Sub-Saharan Africa: A study in Tanzania showcased the efficacy of a cost-effective HCW-led model in bolstering HBV awareness across Sub-Saharan Africa. This innovative approach capitalized on the trust, linguistic compatibility, and close ties local HCWs hold within their communities to effectively disseminate knowledge and tools for HBV awareness [49]. The study underscored the significance of educational resources in facilitating the adoption of strategies such as HBV screening, linkage to care, prevention of MTCT, and prevention of adult acquisition of HBV, mirroring approaches utilized for other infectious diseases like HIV [49].

Knowledge and awareness among young adults in Nigeria: Findings from research conducted in Ekiti, Nigeria, unveiled a notable lack of awareness and knowledge regarding hepatitis B among young adults. The study underscored the imperative of implementing targeted education and vaccination campaigns, mainly through social media platforms, to educate this at-risk population segment and enhance vaccination uptake [50].

Centers for Disease Control and Prevention (CDC)'s know hepatitis B campaign: The CDC initiated the Know Hepatitis B campaign with a specific focus on amplifying testing for HBV among Asian Americans, who bear a disproportionate burden of HBV infection. In collaboration with Hep B United, this campaign aims to heighten awareness, advocate for testing, and promote vaccination to address the health disparity related to HBV within the Asian American community [51]. Employing a multilingual communication strategy, including television and radio public service announcements, online/print advertisements, posters, patient education fact sheets, and engagement across social media platforms, the campaign aims to disseminate critical information about HBV, testing, and vaccination. Additionally, it underscores the importance of community partnerships and localized outreach efforts to educate high-risk communities and foster testing events [51]. These campaigns underscore the importance of tailored educational approaches, community engagement, and targeted interventions in augmenting awareness, knowledge, and testing for hepatitis B, ultimately advancing efforts to mitigate the burden of HBV infection and enhance health outcomes.

## Conclusions

In conclusion, this review has underscored the intricate challenges posed by HBV infection during pregnancy and its profound impact on maternal and neonatal health outcomes. We have illuminated the heightened risk of vertical transmission of HBV from mother to child, emphasizing the potential for chronic HBV infection in offspring and subsequent liver-related complications. Crucially, we have highlighted the imperative of implementing robust screening, diagnostic, and management strategies to avert vertical transmission and mitigate associated risks during pregnancy. Looking ahead, concerted efforts are needed to bolster management and prevention approaches, including the adoption of universal screening protocols for pregnant women, timely access to antiviral therapy for infected mothers, and widespread vaccination of newborns. Moreover, there is a compelling call for intensified public health initiatives aimed at heightening awareness about HBV infection and combating associated stigma. In both clinical practice and public health policy, these findings underscore the necessity for vigilance in screening, counseling, and management of HBV infection during pregnancy. By integrating evidence-based interventions into comprehensive care frameworks, we can strive toward improved outcomes for pregnant women and their newborns affected by HBV infection.
